# The Deubiquitinating Enzyme UBPY Is Required for Lysosomal Biogenesis and Productive Autophagy in *Drosophila*

**DOI:** 10.1371/journal.pone.0143078

**Published:** 2015-11-16

**Authors:** Anne-Claire Jacomin, Amandine Bescond, Emmanuelle Soleilhac, Benoît Gallet, Guy Schoehn, Marie-Odile Fauvarque, Emmanuel Taillebourg

**Affiliations:** 1 Université Grenoble-Alpes, F-38041, Grenoble, France; CEA-DSV-iRTSV-BGE-Gen&Chem, F-38054, Grenoble, France; INSERM, U1038, F-38054, Grenoble, France; 2 Université Grenoble Alpes, IBS, F-38044, Grenoble, France; CNRS, IBS, F-38044, Grenoble, France; CEA, IBS, F-38044, Grenoble, France; IISER-TVM, INDIA

## Abstract

Autophagy is a catabolic process that delivers cytoplasmic components to the lysosomes. Protein modification by ubiquitination is involved in this pathway: it regulates the stability of autophagy regulators such as BECLIN-1 and it also functions as a tag targeting specific substrates to autophagosomes. In order to identify deubiquitinating enzymes (DUBs) involved in autophagy, we have performed a genetic screen in the *Drosophila* larval fat body. This screen identified *Uch-L3*, *Usp45*, *Usp12* and *Ubpy*. In this paper, we show that *Ubpy* loss of function results in the accumulation of autophagosomes due to a blockade of the autophagy flux. Furthermore, analysis by electron and confocal microscopy of *Ubpy*-depleted fat body cells revealed altered lysosomal morphology, indicating that *Ubpy* inactivation affects lysosomal maintenance and/or biogenesis. Lastly, we have shown that shRNA mediated inactivation of UBPY in HeLa cells affects autophagy in a different way: in UBPY-depleted HeLa cells autophagy is deregulated.

## Introduction

Macroautophagy (referred to as ‘autophagy’ hereafter) is the major lysosomal degradation pathway of cytoplasmic components. The overall molecular mechanisms of autophagy are relatively well understood and are conserved in all eukaryotic cells from yeast to humans [1, 2]. The autophagosome formation complex which includes the class III P(I)3-kinase VPS34 and BECLIN-1 initiates the formation of an isolation membrane [3, 4]. Elongation of this membrane then involves two conjugation systems. The first system results in the association of the cytosolic microtubule-associated light-chain 3-I (LC3, also known as Atg8) with phosphatidylethanolamine to generate a lipidated LC3-II form. The second system forms the ATG12-ATG5-ATG16 macromolecular complex. Both conjugation systems contribute to the completion of the double-membraned autophagosomes which eventually fuse with lysosomes to generate the degradative single-membraned autolysosomes. Originally described as a non-specific degradation process limited to bulk cytosol in response to starvation, autophagy is now known to be also responsible for the degradation of specific substrates, including senescent organelles, bacteria, viruses and aggregated proteins (reviewed in refs. [5, 6]).

Ubiquitination is a major post-translational modification which results in the covalent linkage of one or several ubiquitin moieties on substrate proteins. It plays major roles in many cellular processes. In autophagy, it is involved in the regulation of the stability of autophagy regulators such as BECLIN-1 and BCL-2 [7–9]. In addition, ubiquitin functions as a tag targeting specific substrates (protein aggregates, mitochondria or intracellular bacteria) to autophagic degradation [10–12].

Deubiquitinating enzymes (DUBs) remove ubiquitin monomers or polymers from ubiquitinated proteins and thereby serve as key regulators of ubiquitin-dependent processes [13, 14]. A hundred DUBs have been identified in the human genome [15, 16] and the *Drosophila* genome contains 41 DUB encoding genes, 34 of which having at least one human orthologue [17]. Genetic screens identified crucial DUBs involved in the regulation of apoptosis [18], of the Notch pathway [19] and of the innate immune response [20]. DUBs are categorized in five sub-families according to the structure of their catalytic domain: Ubiquitin C-terminal Hydrolases (UCH), Ubiquitin-Specific Proteases (USP), Machado-Joseph Disease Proteases (MJD), Otubain proteases (OTU) and JAB1/MPN/Mov34 Metalloenzymes (JAMM). A few DUBs (all of them belonging the USP class) have been involved in autophagy: Ubp3/Bre5 is required for the starvation-induced degradation of ribosomes by autophagy in yeast [21]; USP15, UBPY and USP30 regulate parkin-mediated mitophagy [22–24]; and USP36 controls selective autophagy activation by ubiquitinated proteins [21, 23–25]. However a systematic analysis of DUBs in autophagy is still lacking.

To identify new DUBs of the USP and UCH sub-families that negatively regulate autophagy *in vivo*, we have systematically silenced by RNAi the corresponding genes in the *Drosophila* larval fat body. This tissue is the primary nutrient storage organ of the larva and produces a robust activation of autophagy in response to nutrient starvation [26]. Moreover, it consists of a monolayer of large, polyploid cells which are ideal for imaging-based techniques [27]. This screen identified four DUBs that may play a role in autophagy: *Uch-L3*, *Usp45*, *Usp12* and *Ubpy*. We further showed that *Uch-L3* and *Usp45* did not act in a cell autonomous manner, whereas *Usp12* and *Ubpy* did. Focusing on *Ubpy*, we have shown that its loss of function results in the accumulation of autophagosomes due to a blockade of the autophagy flux. Furthermore, analysis by electron and confocal microscopy of *Ubpy*-depleted fat body cells revealed altered lysosomal morphology, indicating that *Ubpy* inactivation affects lysosomal maintenance and/or biogenesis. Lastly, we have shown that shRNA mediated inactivation of UBPY in HeLa cells also affects autophagy which appears to be deregulated with an increased number of autophagosomes and increased autophagy flux.

## Results

### A genetic screen for deubiquitinating enzymes involved in autophagy identifies *Ubpy*

**Fig 1:**
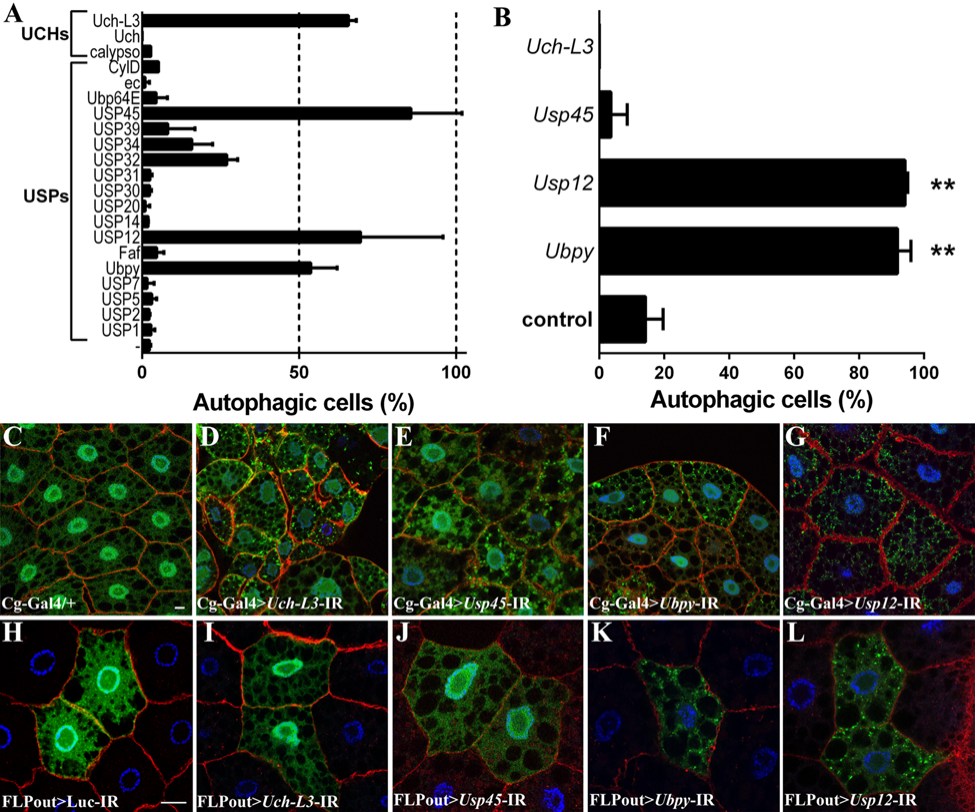
A genetic screen identifies UBPY and USP12 as putative autophagy regulators. (A) Quantification of autophagy in the *Drosophila* larval fat body after silencing of the indicated DUB using the Cg-Gal4 driver line. Bars denote the proportion of autophagic cells from at least 6 animals. Cells were considered as “autophagic” if at least one GFP-LC3B vesicle was observed. (B) Quantification of autophagy after silencing by the FLPout method. Bars denote the proportion of autophagic cells from at least 6 animals. Statistical significance was determined using *one-way ANOVA*: **p<0.005. (C-G) Representative confocal sections after silencing of the indicated DUB in the larval fat body. (H-L) Clonal analysis of the four candidates after silencing by the FLPout method. One representative confocal section per genotype is shown. Actin is labelled with Phalloidin-Texas Red (red) and nuclei are labelled with Hoechst (blue). Scale bar: 10μm. Genotypes: (C) *Cg-Gal4/+; UAS-GFP-LC3B/+*, (D) *Cg-Gal4/ UAS-Uch-L3-IR; UAS-GFP-LC3B/+*, (E) *Cg-Gal4/ UAS-Usp45-IR; UAS-GFP-LC3B/+*, (F) *Cg-Gal4/+; UAS-GFP-LC3B/ UAS-Ubpy-IR*, (G) *Cg-Gal4/+; UAS-GFP-LC3B/ UAS-Usp12-IR*, (H) *y w hs-FLP/+; UAS-GFP-Atg8a/UAS-Luc-IR; Ac>CD2>Gal4/+*, (I) *y w hs-FLP/+; UAS-GFP-Atg8a/UAS-Uch-L3-IR; Ac>CD2>Gal4/+*, (J) *y w hs-FLP/+; UAS-GFP-Atg8a/UAS-Usp45-IR; Ac>CD2>Gal4/+*, (K) *y w hs-FLP/+; UAS-GFP-Atg8a/+; Ac>CD2>Gal4/ UAS-Ubpy-IR*, *(L) y w hs-FLP/+; UAS-GFP-Atg8a/+; Ac>CD2>Gal4/ UAS-Usp12-IR*.

In order to identify new DUBs regulating autophagy, transgenes containing specific inverted repeats (IR) allowing for the production of double stranded RNAs targeting 25 DUBs of the USP and UCH families were expressed in the larval fat body along with the GFP-LC3B autophagic marker using the *Cg-Gal4* driver line [28]. The GFP-LC3B reporter encodes a fusion protein between the Green Fluorescent Protein and the human LC3B protein which is diffused in the cytoplasm and in the nucleus under normal conditions whereas upon autophagy induction, it is recruited onto autophagosomes [29]. In order to identify regulators of autophagy, this screen was carried out on fed mid third-instar larvae that have a low basal level of autophagy (Fig 1A and 1C). Three USPs (*CG4165/Usp45*, *CG5798*/*Ubpy* and *CG7023/Usp12*) and one UCH (*CG3431/Uch-L3*) were retained as candidates for autophagy negative regulators because their silencing induced accumulation of GFP-LC3B positive dots in the cytoplasm of at least 50% of the cells (Fig 1A, 1C–1G and S1 Table). Fat body specific inactivation of three other USPs was lethal at early larval stages and was not further analyzed (S1 Table). The ability of these DUBs to regulate autophagy in a cell-autonomous manner was then tested using the FLPout method [30] to induce RNAi-dependent gene silencing in clones expressing the GFP-Atg8a reporter (Atg8a is a *Drosophila* paralogue of human LC3B) [31]. This clonal analysis revealed that cell-specific silencing of *Uch-L3* and *Usp45* did not result in accumulation of GFP-Atg8a vesicles (Fig 1B, 1I and 1J). As such, these two DUBs may be putative regulators of autophagy at the systemic level, but not at the cellular level and were not further characterized. In contrast, cell-specific inactivation of *Ubpy* and *Usp12* resulted in accumulation of autophagosomes (Fig 1B, 1K and 1L) indicating that Ubpy and Usp12 are putative cell-autonomous regulators of autophagy. We have used a second independent RNAi line targeting *Ubpy* [32] which also resulted in accumulation of autophagosomes (S1 Fig). We have thus focused our investigation on Ubpy because it was not known to play a role in basal autophagy, despite this protein being extensively characterized for its role in endocytosis [33–39] and to a lesser extent in mitophagy [22].

### *Ubpy* inactivation blocks the autophagy flux

**Fig 2:**
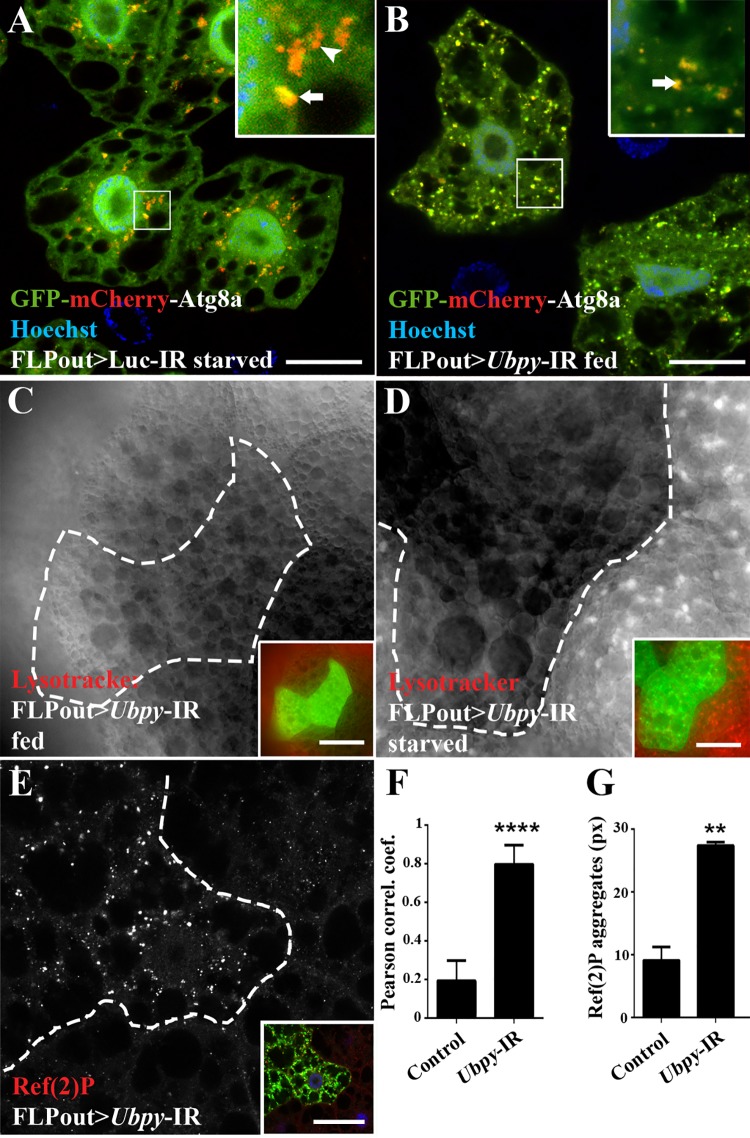
*Ubpy* loss-of-function blocks the autophagy flux. (A,B) Analysis of the autophagy flux using the tandem-tagged GFP-mCherry-Atg8a reporter in control cells from starved larvae (A) or in *Ubpy* silenced cells (B). Insets show an enlarged view for each condition (arrow: autophagosome, arrowhead: autolysosomes). Quantification of the colocalization of mCherry and GFP signals using the Pearson’s correlation coefficient is shown in F. (C,D) Lysotracker Red staining on fat bodies from fed (C) or starved (D) larvae silenced for *Ubpy*. Mutant cells were identified by the expression of the GFP-Atg8a marker (dotted lines and green channel in insets). (E) Confocal sections of larval fat bodies stained for the endogenous Ref(2)P protein. Mutant cells were identified by the expression of the GFP-Atg8a marker (dotted lines and green channel in insets). (G) Quantification of the size of the Ref(2)P aggregates. N>6 larvae per experimental condition. For all the quantifications, bars denote mean ± s.d. Statistical significance was determined using *one-way ANOVA*: *p<0.05, **p<0.005, ****p<0.0001. Scale bars: 20μm (A,B), 50μm (C-E). Genotypes: (A) *y w hs-FLP/+; UAS-GFP-mCherry-Atg8a/UAS-Luc-IR; Ac>CD2>Gal4/+*, (B) *y w hs-FLP/+; UAS-GFP-mCherry-Atg8a/+; Ac>CD2>Gal4/ UAS-Ubpy-IR*, (C-E) *y w hs-FLP/+; UAS-GFP-Atg8a/+; Ac>CD2>Gal4/ UAS-Ubpy-IR*.

An increased number of autophagosomes can either be caused by the activation of the autophagic flux or by the accumulation of basal autophagosomes due to the inhibition of their degradation. To distinguish between these possibilities, we first made use of a transgenic *Drosophila* line expressing the GFP-mCherry-Atg8a fusion protein [40]. This protein produces yellow (green merged with red) fluorescence in autophagosomes and only red fluorescence in autolysosomes due to quenching of the GFP fluorescence in these acidic structures. In fed control larvae expressing this reporter along with a control RNAi transgene targeting Luciferase, no autophagosomes nor autolysosomes were detected (S2 Fig). As expected, control fat body cells in which autophagy has been induced by starvation showed yellow and red vesicles (Fig 2A, S2 Fig) corresponding to autophagosomes and autolysosomes, respectively. In contrast, *Ubpy* mutant fat body cells displayed mainly yellow vesicles (Fig 2B and 2F and S2 Fig), indicating the presence of autophagosomes but lack of autolysosomes. Lysotracker staining is another established and widely used assay for detecting acidic compartments such as lysosomes and autolysosomes [26, 41]. In fed mid-third instar larvae we did not observe any accumulation of Lysotracker positive vesicles in the mutant cells (Fig 2C), despite our previous results showing that *Ubpy* mutant cells accumulate autophagosomes. Furthermore, starvation induced Lysotracker staining [26] was strongly suppressed in *Ubpy* mutant cells compared to wild-type neighboring cells (Fig 2D), indicating that *Ubpy* inactivation also impaired starvation induced autophagy. The same result is observed using the GFP-mCherry-Atg8a reporter in starved *Ubpy* silenced cells (S2 Fig). The autophagy flux can also be monitored by assessing the degradation of known autophagic substrates such as the p62 protein, which accumulates upon autophagy flux blockade [42, 43]. Using a specific antibody raised against Ref(2)P, the *Drosophila* p62 homolog [44], we observed the accumulation of the Ref(2)P/p62 protein in *Ubpy* silenced cells compared to wild-type neighboring cells (Fig 2E and 2G). Altogether, these results unambiguously demonstrate that *Ubpy* inactivation results in a blockade of the autophagic flux.

**Fig 3:**
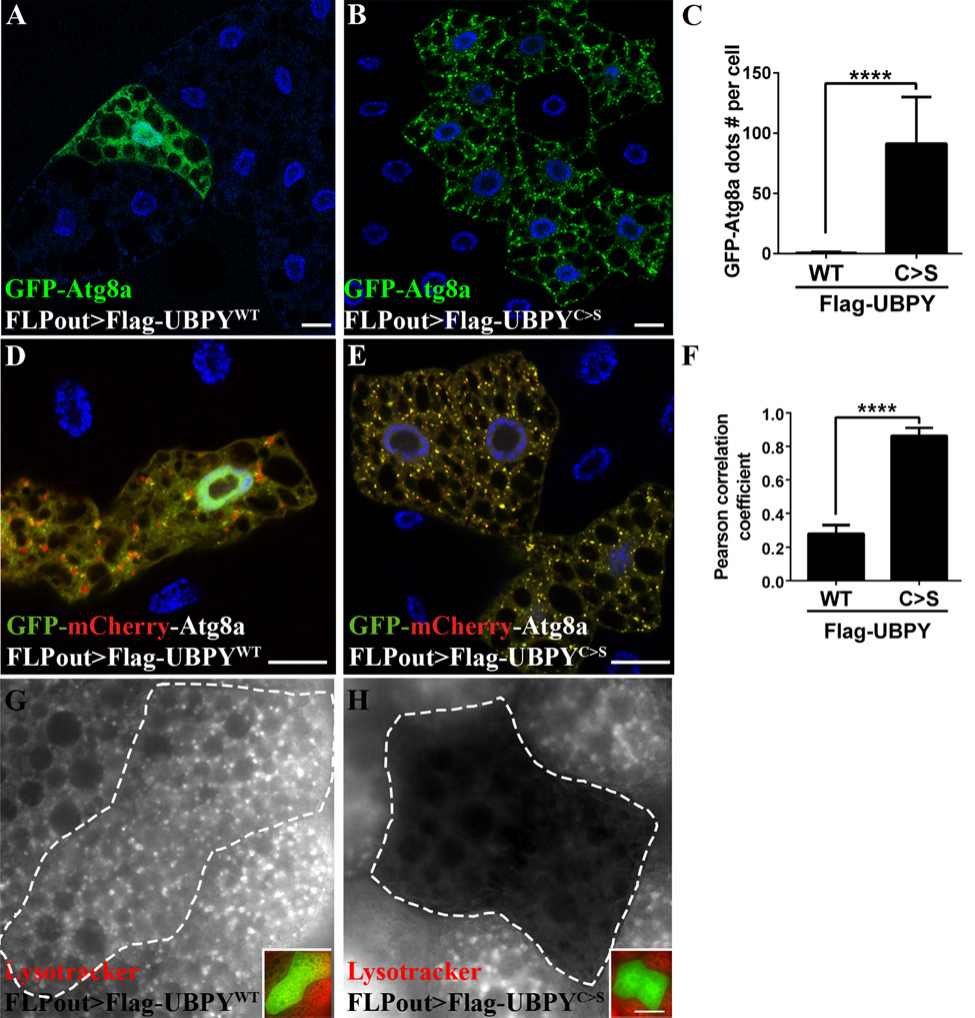
Expression of a catalytic inactive UBPY mutant blocks the autophagy flux. Expression of the wild-type form of UBPY has no effect on autophagy (Flag-UBPY^WT^, A) whereas the UBPY catalytic mutant form induces accumulation of GFP-Atg8a dots (Flag-UBPY^C>S^, B). Quantification of the number of GFP-Atg8a dots per cell is show in C. Confocal sections of larval fat bodies expressing the GFP-mCherry-Atg8a in combination with the wild-type (D) or catalytic inactive (E) forms of UBPY. Please note that the expressing the wild-type form of UBPY (D) were starved to induce autophagy and allow the observation of autophagosomes. (F) Quantification of the colocalization of mCherry and GFP signals using the Pearson’s correlation coefficient. Larvae expressing the wild-type (G) or the mutant (H) form of UBPY were starved to induce autophagy and stained with Lysotracker Red. Insets show the merged channels of the respective images and the clone boundaries are indicated as dotted lines. Scale bars: 20μm. N>6 larvae per experimental condition. For quantification, bars denote mean ± s.d. Statistical significance was determined using *one-way ANOVA*: ****p<0.0001, ns: not significant. Genotypes: (A, G) *y w hs-FLP/+; UAS-GFP-Atg8a/+; Ac>CD2>Gal4/UAS-2xFlag-UBPY* ^*WT*^, (B, H) *y w hs-FLP/+; UAS-GFP-Atg8a/+; Ac>CD2>Gal4/ UAS-2xFlag-UBPY* ^*C>S*^, (D) *y w hs-FLP/+; UAS-GFP-mCherry-Atg8a/+; Ac>CD2>Gal4/UAS-2xFlag-UBPY* ^WT^, (E) *y w hs-FLP/+; UAS-GFP-mCherry-Atg8a/+*,*; Ac>CD2>Gal4/ UAS-2xFlag-UBPY* ^*C>S*^.

We then made use of two transgenic lines expressing either the Flag tagged wild-type UBPY protein (Flag-UBPY^WT^) or its catalytically inactive, dominant negative counterpart (Flag-UBPY^C>S^) [45]. Expression of the Flag-UBPY^C>S^ protein resulted in the accumulation of GFP-Atg8a positive autophagosomes whereas expression of the wild-type protein had no effect (Fig 3A–3C). Analysis of the GFP-mCherry-Atg8a distribution in starved Flag-UBPY^WT^ expressing cells showed that both yellow and red dots were observed indicating that autophagosomes and autolysosomes were present (Fig 3D and 3G), consistent with an increase of the autophagic flux in response to starvation. By contrast, in Flag-UBPY^C>S^ expressing cells, we observed mostly yellow dots and no red dots indicating the presence of autophagosomes and absence of autolysosomes (Fig 3E and 3G). Lastly, Lysotracker staining of starved third-instar larvae fat bodies showed that Flag-UBPY^WT^ expressing cells (Fig 3G) were not different from wild-type neighboring cells whereas a nearly complete loss of Lysotracker staining was observed in Flag-UBPY^C>S^ expressing cells (Fig 3H). These results show that expression of the dominant negative form of UBPY results in a blockade of the autophagic flux, mimicking the phenotype induced by RNAi. In addition to validating the specificity of the *Ubpy*-targeting RNAi transgenes, these results further indicate that the deubiquitinating activity of UBPY is required to maintain the autophagic flux.

### *Ubpy* inactivation induces lysosomal defects

**Fig 4:**
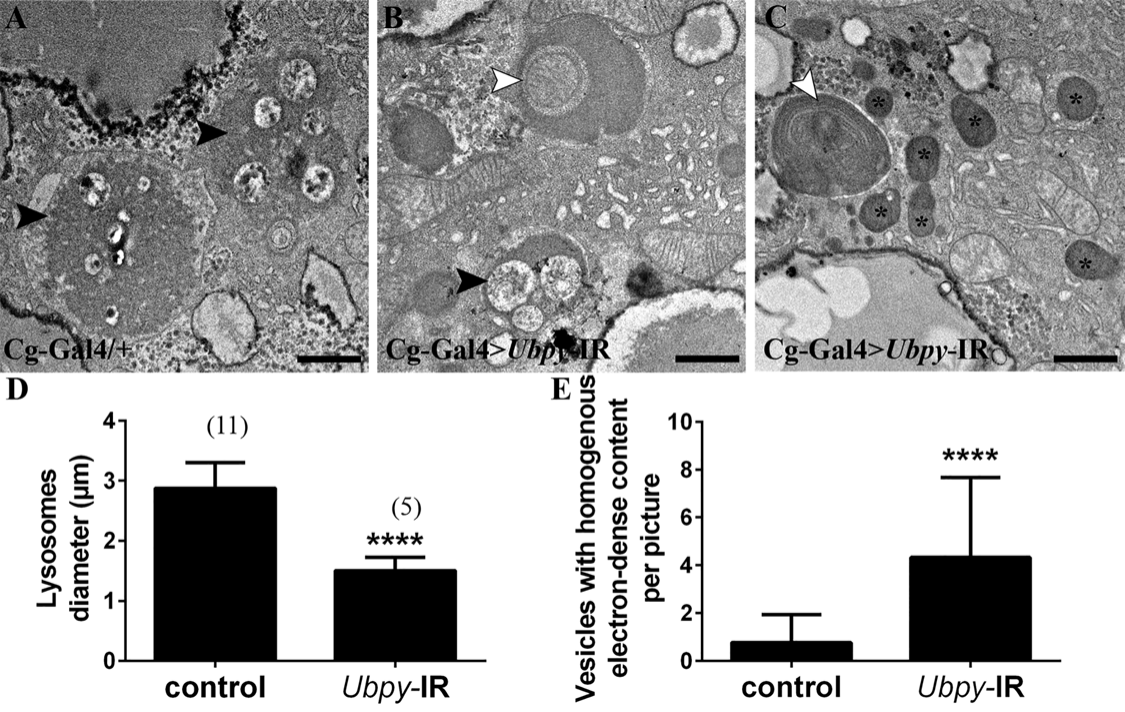
Ultrastructural analysis of *Ubpy* silenced cells. Control fat body cells (A) contain large autolysosomes (black arrowhead). These vesicles are characterized by their heterogeneous content and organelle remnants. In contrast, *Ubpy* silenced cells (B-C) contain autophagosomes whith non-degraded organelles (white arrowhead) (a mitochondria in B and endoplasmic reticulum in C), small autolysosomes (black arrowhead) and vesicles with homogenous electron-dense content (asterisks). Scale bars: 1μm. (D-E) Quantification of lysosomal diameter (D) and number of vesicles with homogenous electron-dense content (E). Bars denote mean ± s.d. Statistical significance was determined using *t-test*: ****p<0.0001. Genotypes: (A) *Cg-Ggal4/+*, (B-C) *Cg-Gal4/+; UAS-Ubpy-IR/+*.

To get further insight into the role of UBPY in autophagy, we turned to ultrastructural analysis by electron microscopy. In wild-type larvae rare degradative lysosomes with cytoplasmic components at various stages of degradation were present (Fig 4A and refs. [29, 46]). In *Ubpy* silenced fat body cells, double membrane autophagosomes with non-degraded content were present, which is consistent with our previous results showing a blockade of the autophagic flux (Fig 4B and 4C). We further observed significantly smaller lysosomes and numerous small vesicles with homogenous, electron-dense contents corresponding to vesicles budding from the Golgi apparatus and transporting lysosomal hydrolases to late endosomes (Fig 4B–4E). These observations fully support the view that *Ubpy* loss-of-function in *Drosophila* fat body cells results in the accumulation of autophagosomes and further indicate morphological defects in lysosomes of *Ubpy* inactivated cells.

**Fig 5:**
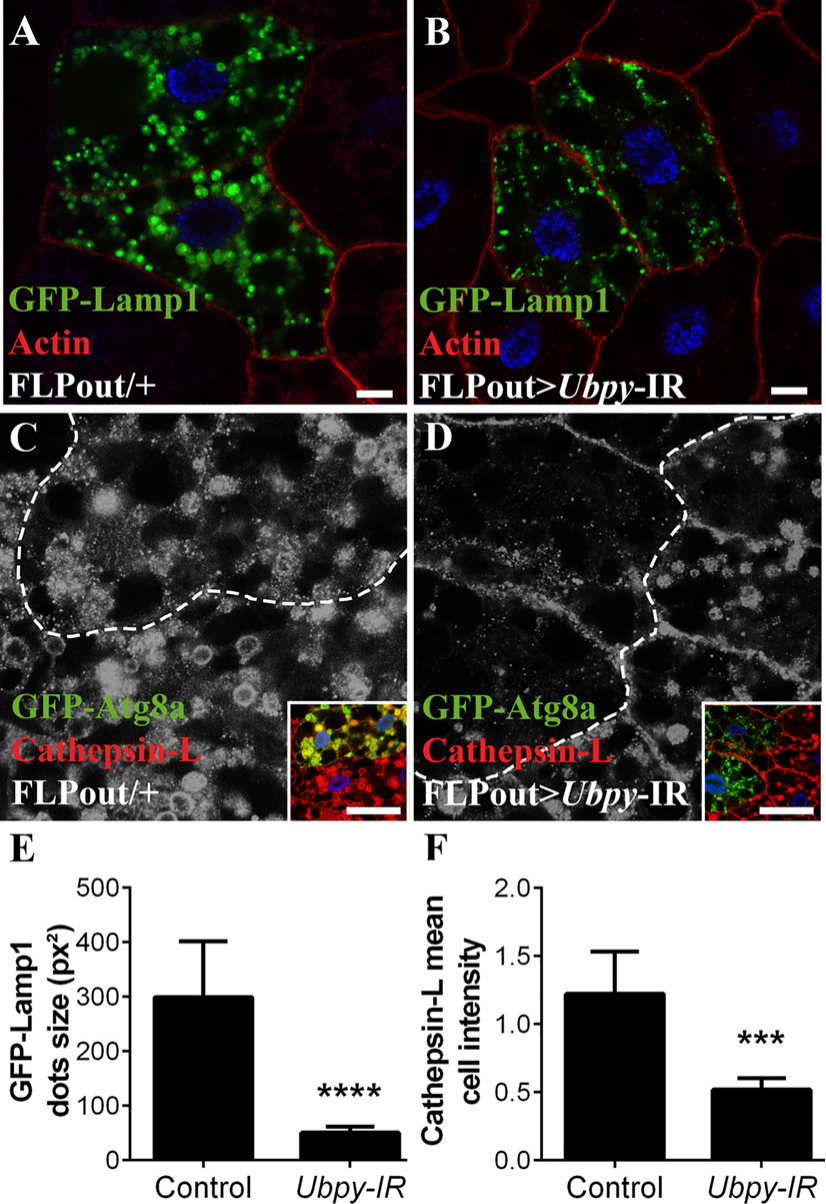
*Ubpy* silencing induces lysomal defects. (A,B) Confocal sections of larval fat bodies clonally expressing the lysosomal markers GFP-Lamp1 alone (A) or in combination with the *Ubpy* silencing transgene (B). (C,D) Confocal sections of larval fat bodies clonally expressing the autophagy reporter GFP-Atg8a alone (C) or in combination with the *Ubpy* silencing transgene (D) after staining for the endogenous lysosomal hydrolase Cathepsin-L. Insets show the merged channels of the respective images and the clone boundaries are indicated as dotted lines (E). Quantification of GFP-Lamp1 dots size. (F) Quantification of the mean relative intensity of the Cathepsin-L staining in GFP-Atg8a expressing cells compared to the staining intensity of the adjacent wild-type neighboring cells. N>6 larvae per experimental condition. Bars denote mean ± s.d. Statistical significance was determined using *one-way Anova*: *p<0.05, **p<0.005, ***p<0.0005, ****p<0.0001. Scale bar: 10μm (A-H), 50μm (J-Q). Genotypes: (A) *y w hs-FLP/+; UAS-GFP-Lamp1/+; Ac>CD2>Gal4/+*, (B) *y w hs-FLP/+; UAS-GFP-Lamp1/+; Ac>CD2>Gal4/UAS-Ubpy-IR*, (C) *y w hs-FLP/+; UAS-GFP-Atg8a/+; Ac>CD2>Gal4/+* (D) *y w hs-FLP/+; UAS-GFP-Atg8a/+; Ac>CD2>Gal4/ UAS-Ubpy-IR*.

In order to be degraded autophagososmes have to fuse with lysosomes. Thus any disturbance of the function and/or biogenesis of lysosomes could potentially affect autophagy [47]. Two classes of proteins are essential for the function of lysosomes: soluble lysosomal hydrolases (also known as acid hydrolases) and integral lysosomal membrane proteins (LMP). One of the most abundant LMP is the lysosomal-associated membrane protein 1 (LAMP1). In wild-type cells, a GFP-LAMP1 fusion protein stained large perinuclear vesicles corresponding to lysosomes as well as smaller vesicles distributed in the cytoplasm (Fig 5A). In cells expressing the dsRNA targeting *Ubpy*, the large perinuclear lysosomes were missing whereas smaller dots were still present (Fig 5B and 5E). These results thus show that inactivation of *Ubpy* results in a marked reduction of the size of lysosomes as visualized by the GFP-LAMP1 marker.

As previously indicated lysosomes contain many acid hydrolases which are responsible for their catabolic capacity and cathepsins are important constituents of this lytic system. These enzymes are synthesized in the endoplasmic reticulum, sorted in the Golgi apparatus using the mannose-6-phosphate receptor and delivered to late endosomes [48]. In starved wild-type larvae, Cathepsin L staining identifies large lysosomes (Fig 5C). In contrast, cells expressing the dsRNA targeting *Ubpy* showed a drastic change in Cathepsin L distribution: the overall staining intensity was decreased (Fig 5D and 5F), the large lysosomes were missing and only dots presumably corresponding to vesicles transporting Cathepsin L from the Golgi apparatus were present. Combined with our previous data, these results thus indicate that inactivation of *Ubpy* strongly affects lysosomal morphology and/or biogenesis.

### Inactivation of human UBPY in HeLa cells activates autophagy

**Fig 6:**
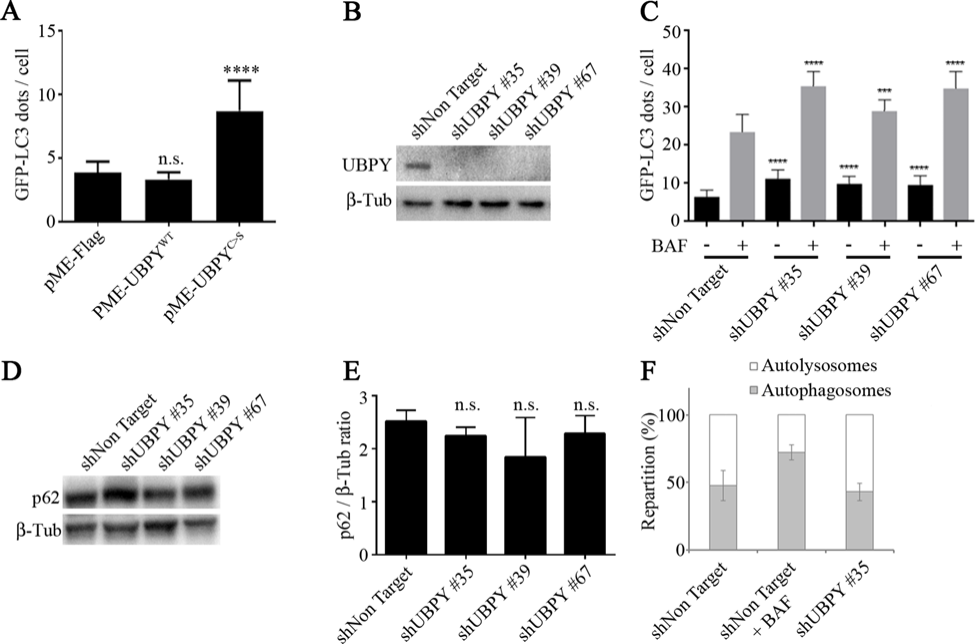
UBPY silencing in HeLa cells activates autophagy. (A) The number of GFP-LC3 dots per cell was quantified in HeLa cells stably expressing the autophagy reporter GFP-LC3; cells were transfected with a control plasmid (pME-Flag) or plasmids expressing either the wild-type human UBPY protein (pME-UBPY^WT^) or its catalytically inactive mutant (pME-UBPY^C>S^). Bars denote mean ± s.d. Statistical significance was determined using *t-test*: ****p<0.0001 (B) The expression of UBPY was monitored by Western blot in GFP-LC3 HeLa cells stably transfected with a control shRNA or three different shRNAs targeting UBPY. (C) The number of GFP-LC3 dots per cell was quantified in GFP-LC3 HeLa cells stably transfected with a control shRNA or three different shRNAs targeting UBPY in absence (black bars) or in presence of bafilomycin A1 (BAF, gray bars). Bars denote mean ± s.d. Statistical significance was determined using *t-test*: ****p<0.0001; ***p<0.005 (D) The expression of the autophagy target protein p62 was monitored by Western blot in GFP-LC3 HeLa cells stably transfected with a control shRNA or three different shRNAs targeting UBPY. (E) Quantification of p62 levels in GFP-LC3 HeLa cells stably transfected with a control shRNA or three different shRNAs targeting UBPY from three independent Western blots. (F) The repartition of autolysosmes and autophagosomes was determined in mRFP-GFP-LC3 HeLa cells stably expressing either the control shRNA or the shUBPY #35 shRNA, in comparison with control transfected cells treated with bafilomycin A1.

In human cells, UBPY has been shown to regulate mitophagy (elimination of damaged mitochondria by autophagy) by controlling Parkin recruitment to depolarized mitochondria after CCCP treatment [22]. However, its role in basal autophagy has not been assessed. We first overexpressed the wild-type UBPY protein and its catalytically inactive, dominant negative mutant form [32] in HeLa cells stably expressing the GFP-LC3 autophagy reporter [49]. Whereas overexpression of wild-type UBPY had no effect, overexpression of its catalytically inactive mutant form significantly increased the number of autophagosomes per cell (Fig 6A, S3 Fig). We then established GFP-LC3 HeLa cell lines stably expressing either a control shRNA (shNon Target) or three different shRNAs targeting UBPY (see Material and Methods for details). Compared to control the three shUBPY cell lines showed a drastic reduction of the UBPY protein as determined by Western blot (Fig 6B). These UBPY-depleted cell lines showed a significant increase of the number of autophagosomes (Fig 6C, S4 Fig).

We next asked whether autophagosomes accumulation in UBPY knock-down cells is the result of *de novo* autophagosome formation due to autophagy induction or of inhibition of their degradation blocking the autophagy flux. First, we analyzed autophagy in the presence of bafilomycin A1 (BAF), an inhibitor of autolysosome acidification. We found that BAF treatment further increased the number of GFP-LC3 dots observed in shUBPY expressing cells as compared to shNon Target expressing cells (Fig 6C, S4 Fig). This result suggests that the observed accumulation of autophagosomes in UBPY knocked down cells is not due to a full autophagy flux blockade. We further observed that the levels of the autophagy target protein p62 were not different in shUBPY expressing cells compared to control cells (Fig 6D and 6E) which also indicates that the autophagy flux is not stalled in UBPY knocked down cells. Finally, we used HeLa cells stably expressing the mRFP-GFP-LC3 reporter that allows for the distinction between autophagosomes (GFP^+^RFP^+^) and autolysosomes (GFP^−^RFP^+^) due to the quenching of the GFP signal in acidic compartments [50]. We established mRFP-GFP-LC3 HeLa cell lines stably expressing either the control shNon Target shRNA or the shUBPY #35 shRNA. In keeping with our previous results we found an increased number of total autophagy vesicles in UBPY inactivated cells or in control cells treated with bafilomycin A1 (S5 Fig). Furthermore, we observed an equivalent ratio of autophagosomes and autolysosomes in shNon Target and shUBPY expressing cells, which is in contrast with the increased proportion of autophagosomes to autolysosomes detected in bafilomycin A1 treated control cells (Fig 6F). Taken altogether, these results show that UBPY knock-down in HeLa cells results in a deregulation of the autophagy flux.

## Discussion

In order to identify new DUBs involved in autophagy, the 25 DUBs of the USP and UCH sub-families were screened by RNAi for the induction of an autophagic phenotype in the *Drosophila* larval fat body. *Uch-L3*, *Usp45*, *Usp12* and *Ubpy* were identified in this screen and additional experiments demonstrated that only *Usp12* and *Ubpy* are involved in autophagy in a cell-autonomous manner while the two other DUBs may regulate autophagy at the systemic level. Focusing on *Ubpy*, we have shown that its silencing using two different RNAi transgenes as well as the expression of a dominant negative UBPY protein blocked the autophagy flux. Ultrastructural analysis of *Ubpy* mutant fat bodies confirmed the presence of autophagosomes with non-degraded contents and further showed that lysosomes were significantly smaller than those observed in wild-type cells. We then looked at lysosomal markers such as the lysosomal membrane protein LAMP1 and the lysosomal hydrolase Cathepsin L and observed that *Ubpy* silencing resulted in severe lysosomal defects. Taken altogether, these results show that in the *Drosophila* fat body, UBPY is required for lysosomal biogenesis and/or maintenance and strongly suggest that, as a consequence, the lysosomal defects induced by *Ubpy* silencing affects autophagic degradation. We have then asked whether the human UBPY protein plays a role in the regulation of basal autophagy. We have observed that overexpression of a dominant negative human UBPY protein or silencing of UBPY in HeLa cells also affected autophagy but by actvating it rather than by blocking the autophagy flux as observed in *Drosophila*.

In light of the different results obtained in human cells and *Drosophila*, we cannot rule out the possibility that in *Drosophila*, UBPY silencing results in both autophagy activation and degradation defects (with the latter being the only observable one) whereas in HeLa cells, UBPY silencing did not result in a full blockade of autophagy, allowing to detect an additional role in autophagy regulation. Alternatively, as the DUB complement of mammals is much more important than the one of *Drosophila* (approximately 100 DUBs in the human genome [15, 16] *versus* 41 in the *Drosophila* genome [17]), some redundancy may exist and another human DUB may fulfill its function in lysosomal biogenesis.

In human, in addition to its role in endocytosis UBPY has been shown to regulate mitophagy by controlling Parkin recruitment to depolarized mitochondria after CCCP treatment [22]. We show here that UBPY silencing also activates autophagy in absence of CCCP treatment, which strongly suggests that UBPY is not connected exclusively to mitophagy. Additional experiments will be necessary to thoroughly characterize the role of UBPY in this process.

UBPY is mainly known to act in endocytosis. In *Drosophila*, it controls the stability of the ESCRT-0 subunit Hrs and deubiquitinates cargo of the endocytic pathway. Moreover, its inactivation results in major defects in the endocytic pathway with the accumulation of enlarged endosomes enriched in signaling molecules [33]. It has also been shown to regulate the intracellular trafficking of signaling molecules of the *Hedghog* and *Frizzled* pathways [32, 45, 51]. A tempting hypothesis is that the defects in lysosomes biogenesis and/or maintenance that we have observed in *Ubpy*-depleted fat body cells, are related to these defects in the endocytic pathway. Alternatively, UBPY has also been shown to deubiquitinate proteins which are not part of the endocytic machinery such as the TDP-43 and CLOCK proteins [52, 53]. It is possible that UBPY has additional, yet unknown substrates that would account for the lysososomal defects observed in *Ubpy* mutant cells. Our work thus open new avenues to the different roles of UBPY and future work will be needed to get a clear understanding of UBPY functions.

## Materials and Methods

### Drosophila stocks and clonal analysis

Flies were reared at 25°C on standard cornmeal–yeast medium. The RNAi transgenes targeting the DUBs were obtained from the Vienna Drosophila Resource Center. The second independant RNAi line targeting *Ubpy* was obtained from Dr. Goto [32]. The *UAS-2xFlag-UBPY* strains were obtained from Dr. Jia [45]. The *UAS-Luc-IR* and *UAS-GFP-mCherry-Atg8a* (#37749) strains were obtained from the Bloomington *Drosophila* Stock Center. The *UAS-GFP-Atg8a* and *UAS-GFP-LC3B*lines were obtained from Dr. T. Neufeld and Dr. H. Stenmark, respectively. For the FLPout Gal4/UAS method, spontaneous activation of the Gal4 transcription factor has been reported and allows for the induction of Gal4 expressing cells without heat shock [31].

### Immunocytochemistry and microscopy

Antibody and phalloidin stainings were performed as described previously [25]. The samples were imaged with a 63x magnification (oil immersion) using a Leica TCS-SP2 confocal microscope and the LCS software. The primary antibodies used in this study were the following: rabbit polyclonal against *D*. *melanogaster* Ref(2)P protein [54], mouse monoclonal against Flag tag (Clone M2, Sigma-Aldrich) and rabbit monoclonal anti-Cathepsin L (ab133641, Abcam). The appropriate Cy3-conjugated secondary antibodies were purchased from Jackson Immunoresearch Laboratories.

Lysotracker staining on tissue was performed as in ref. [55]. Images were obtained with a fluorescence microscope (Nikon Eclipse 90i) controlled by Nikon Software (Universal Imaging Corp.) using a 60x Plan-Neofluor oil objective.

Image analysis and processing were done with Fiji/ImageJ (National Institute of Health) and Photoshop CS6 (Adobe).For experiments carried out in HeLa cells, the following antibodies were used: anti-β-tubulin monoclonal antibody (Sigma-Aldrich), anti-p62 monoclonal antibody (H00008878-M01, Novus Biologicals), anti-UBPY. Dimethyl sulfoxyde (DMSO) and Hoechst #33342 were from Sigma-Aldrich and Bafilomycin A1 (#tlrl-baf1) was purchased from Invivogen.

### Electron microscopy

Fed or starved mid-third instar larvae were dissected in PBS. The inverted carcasses were fixed for 2h at room temperature in 2% paraformaldehyde, 0.2% glutaraldehyde in 0.1M cacodylate buffer pH 7.2, rinsed in 0.1M cacodylate buffer, and post-fixed in 1% OsO4, 1.5% potassium ferrocyanide in 0.1M cacodylate buffer for 1h at 4°C. After washes in water, post-staining was done using 5% uranyl-acetate in water for 1h at room temperature in the dark. Carcasses were then dehydrated in graded ethanol series, and dissected fat bodies were embedded in EMBed812 (EMS, 14120), 0,2% DMP30. Ultrathin sections (80nm) were cut using a Leica UC7 ultra-microtome and DiATOME 35° diamond knife and collected on formvar carbon coated 100mesh grids. Sections were stained in 5% uranyl acetate (in water) for 5 minutes and in 2% lead citrate for 5 additional minutes. Images were taken with a CM12 Philips electron microscope at 120 kV using an ORIUS SC1000 CCD camera (Gatan).

### Starvation experiments

Feeding larvae were washed twice in PBS and starved for 4 hours on 20% sucrose as an amino-acids-deficient starvation medium [26].

### Cells

Cells were maintained in RPMI (for the GFP-LC3 HeLa cells [49]) or DMEM medium (for the mRFP-GFP-LC3 HeLa cells [50]) supplemented with 10% fetal bovine serum, 0.5 mg/ml G418 and 1% penicillin/streptomycin. Plasmids were transfected with Fugene (Promega) 48 hrs prior analysis.

### Lentiviral shRNA transduction

Lentiviral particles were from the Sigma-Aldrich MISSION^®^ shRNA library; each shRNA was inserted in a pLKO.1-PURO plasmid (shNon Target, shUBPY #35, shUBPY #39 and shUBPY #67).

Cells were seeded in 96-well plates (#655090, Greiner) at 12000 cells per well in RPMI. After 18 hours, the medium of each well was replaced with RPMI containing 8μg/ml polybren (Sigma-Aldrich). Lentiviral particles were added at a M.O.I of 5 and 72 hours later, 1μg/ml puromycin (Sigma-Aldrich) was added.

### Autophagy quantification in HeLa cells

96-well plates (#655090, Greiner) were seeded at 7500 cells/well. Twenty-four hours later, cells were treated with 0.1μM Bafilomycin A1or 0.5% DMSO for 2h. The cells were then washed with PBS1X, fixed with 4% paraformaldehyde for 15 minutes, washed twice with PBS 1X and DNA was stained with 1 μg/ml Hoechst (#33342, Sigma-Aldrich) for 30 minutes. Cells were rinsed three times with PBS1X/Tween-20 0.1% and wells filled with PBS1X/Glycerol 50%.

The image acquisitions were performed on an automated microscope ArrayScan^VTI^ (Thermo Scientific) using a Zeiss 20x Plan-Neofluor air objective. Ten fields (corresponding approximately to 1000 cells) were systematically acquired for each fluorescent channels.

Quantification of autophagosomes and autolysosomes was made using to the SpotDetector Bio-Application of Thermo Scientific HCS Studio v6.5.0. Briefly, each nucleus was detected in the Hoechst channel. GFP negative cells were eliminated from the GFP-LC3 channel and cytoplasmic GFP^+^ dots were counted allowing the extraction of the GFP-LC3 dots/cell parameter in the case of GFP-LC3 HeLa cell line. For the mRFP-GFP-LC3 HeLa cells, once the nuclei mask was obtained, RFP+ dots were counted first. Then the RFP mask was applied to the GFP channel allowing for the distinction between autophagosomes which are GFP^+^RFP^+^ and autolysosomes which are GFP^-^RFP^+^. The results were the mean of three different experiments performed in triplicates.

### Statistical analysis

Statistical analyses were performed using Prism 6 (GraphPad). For the comparison of two groups, *t test* has been used. To compare three or more groups, *one-way ANOVA* with the *Dunnett’s test* for multiple comparisons have been used.

## Supporting Information

S1 Fig A second independant RNAi line targeting *Ubpy* results in autophagosomes accumulation. (A) Clonal analysis of a second independant RNAi line targeting *Ubpy* (Mukai et al 2010) using the FLPout method. Actin is labelled with Phalloidin-Texas Red (red) and nuclei are labelled with Hoechst (blue). Scale bar: 10μm. (B) Quantification of autophagy after silencing by the FLPout method. Bars denote the proportion of autophagic cells from at least 6 animals. Statistical significance was determined using *one-way ANOVA*: **p<0.005. Genotypes: (A) *y w hs-FLP/+; UAS-GFP-Atg8a/+; Ac>CD2>Gal4/ UAS-Ubpy-IR2*.
(PDF)

S2 Fig *Ubpy* loss-of-function blocks the autophagy flux. Analysis of the autophagy flux using the tandem-tagged GFP-mCherry-Atg8a reporter in control larvae (A,B) or in *Ubpy* silenced cells (C-F). Quantification of the colocalization of mCherry and GFP signals using the Pearson’s correlation coefficient is shown in G. N>6 larvae per experimental condition. For all the quantifications, bars denote mean ± s.d. Statistical significance was determined using *one-way ANOVA*: *p<0.05, **p<0.005, ****p<0.0001. Genotypes: (A, B) *y w hs-FLP/+; UAS-GFP-mCherry-Atg8a/UAS-Luc-IR; Ac>CD2>Gal4/+*, (C, D) *y w hs-FLP/+; UAS-GFP-mCherry-Atg8a/+; Ac>CD2>Gal4/ UAS-Ubpy-IR*, (E, F) *y w hs-FLP/+; UAS-GFP-mCherry-Atg8a/+; Ac>CD2>Gal4/ UAS-Ubpy-IR2*.
(PDF)

S3 Fig UBPY interferes with autophagy in HeLa cells. HeLa cells stably expressing the autophagy reporter GFP-LC3 were transfected with a control plasmid (pME-Flag, A) or plasmids expressing either the wild-type human UBPY protein (pME-UBPY^WT^, B) or its catalytically inactive mutant (pME-UBPY^C>S^, C).
(PDF)

S4 Fig UBPY silencing interferes with autophagy in HeLa cells. GFP-LC3 HeLa cells were stably transfected with a control shRNA (A, E) or three different shRNAs targeting UBPY (B-D, F-G) in absence (A-D) or in presence of bafilomycin A1 (BAF, E-H).
(PDF)

S5 Fig UBPY silencing activates autophagy in HeLa cells. mRFP-GFP-LC3 HeLa cells were stably transfected with either the control shRNA (A, B) or the shUBPY #35 shRNA (C), in comparison with control transfected cells treated with bafilomycin A1 (B). mRFP (A-C), GFP (A’-C’), merge (A”-C”). (D) Quantification of autophagosomes and autolysosomes.
(PDF)

S1 Table Phenotypes induced by the silencing of the indicated DUB in the larval fat body. List of the phenotypes associated with the fat body specific silencing (using the *Cg-GAL4* driver) of the USPs and UCHs tested in this study. Please note that the DUB encoded by the gene CG5505 also known as *scrawny* or *dUsp36* has not been included in this study because its role in autophagy has already been characterized [25].
(PDF)

## References

[1] Mulakkal NC, Nagy P, Takats S, Tusco R, Juhász G, Nezis IP. Autophagy in Drosophila: From Historical Studies to Current Knowledge. BioMed Research International. 2014;2014:24. 10.1155/2014/273473 PMC4052151 24949430

[2] Yang Z, Klionsky DJ. Mammalian autophagy: core molecular machinery and signaling regulation. Current opinion in cell biology. 2010;22(2):124–31. 10.1016/j.ceb.2009.11.014 20034776 PMC2854249

[3] Liang XH, Jackson S, Seaman M, Brown K, Kempkes B, Hibshoosh H, et al Induction of autophagy and inhibition of tumorigenesis by beclin 1. Nature. 1999;402(6762):672–6. 10604474 10.1038/45257

[4] Klionsky DJ, Codogno P. The mechanism and physiological function of macroautophagy. Journal of innate immunity. 2013;5(5):427–33. 10.1159/000351979 23774579 PMC6741458

[5] Shaid S, Brandts CH, Serve H, Dikic I. Ubiquitination and selective autophagy. Cell Death Differ. 2013;20(1):21–30. 10.1038/cdd.2012.72 22722335 PMC3524631

[6] Johansen T, Lamark T. Selective autophagy mediated by autophagic adapter proteins. Autophagy. 2011;7(3):279–96. 21189453 10.4161/auto.7.3.14487 PMC3060413

[7] Shi C-S, Kehrl JH. Traf6 and A20 differentially regulate TLR4-induced autophagy by affecting the ubiquitination of Beclin 1. Autophagy. 2010;6(7):986–7. 10.4161/auto.6.7.13288 20798608 PMC3039745

[8] Platta HW, Abrahamsen H, Thoresen SB, Stenmark H. Nedd4-dependent lysine-11-linked polyubiquitination of the tumour suppressor Beclin 1. Biochem J. 2012;441(1):399–406. 10.1042/BJ20111424 21936852 PMC3242507

[9] Chen D, Gao F, Li B, Wang H, Xu Y, Zhu C, et al Parkin mono-ubiquitinates Bcl-2 and regulates autophagy. J Biol Chem. 2010;285(49):38214–23. 10.1074/jbc.M110.101469 20889974 PMC2992255

[10] Kaniuk NA, Kiraly M, Bates H, Vranic M, Volchuk A, Brumell JH. Ubiquitinated-protein aggregates form in pancreatic beta-cells during diabetes-induced oxidative stress and are regulated by autophagy. Diabetes. 2007;56(4):930–9. 17395740 10.2337/db06-1160

[11] Thurston TLM, Ryzhakov G, Bloor S, von Muhlinen N, Randow F. The TBK1 adaptor and autophagy receptor NDP52 restricts the proliferation of ubiquitin-coated bacteria. Nat Immunol. 2009;10(11):1215–21. 10.1038/ni.1800 19820708

[12] Beau I, Esclatine A, Codogno P. Lost to translation: when autophagy targets mature ribosomes. Trends Cell Biol. 2008;18(7):311–4. 10.1016/j.tcb.2008.05.001 18508269

[13] Clague MJ, Barsukov I, Coulson JM, Liu H, Rigden DJ, Urbe S. Deubiquitylases from genes to organism. Physiol Rev. 2013;93(3):1289–315. 10.1152/physrev.00002.2013 23899565

[14] Eletr ZM, Wilkinson KD. Regulation of proteolysis by human deubiquitinating enzymes. Biochim Biophys Acta. 2014;1843(1):114–28. 10.1016/j.bbamcr.2013.06.027 23845989 PMC3833951

[15] Nijman SM, Luna-Vargas MP, Velds A, Brummelkamp TR, Dirac AM, Sixma TK, et al A genomic and functional inventory of deubiquitinating enzymes. Cell. 2005;123(5):773–86. 16325574 10.1016/j.cell.2005.11.007

[16] Hutchins AP, Liu S, Diez D, Miranda-Saavedra D. The repertoires of ubiquitinating and deubiquitinating enzymes in eukaryotic genomes. Mol Biol Evol. 2013;30(5):1172–87. 10.1093/molbev/mst022 23393154 PMC3670738

[17] Tsou W-L, Sheedlo MJ, Morrow ME, Blount JR, McGregor KM, Das C, et al Systematic analysis of the physiological importance of deubiquitinating enzymes. PLoS One. 2012;7(8):e43112 10.1371/journal.pone.0043112 22937016 PMC3427330

[18] Broemer M, Tenev T, Rigbolt KT, Hempel S, Blagoev B, Silke J, et al Systematic in vivo RNAi analysis identifies IAPs as NEDD8-E3 ligases. Mol Cell. 2010;40(5):810–22. 10.1016/j.molcel.2010.11.011 21145488

[19] Zhang J, Liu M, Su Y, Du J, Zhu AJ. A targeted in vivo RNAi screen reveals deubiquitinases as new regulators of Notch signaling. G3 (Bethesda). 2012;2(12):1563–75. 23275879 10.1534/g3.112.003780 PMC3516478

[20] Engel E, Viargues P, Mortier M, Taillebourg E, Coute Y, Thevenon D, et al Identifying USPs regulating immune signals in Drosophila: USP2 deubiquitinates Imd and promotes its degradation by interacting with the proteasome. Cell Commun Signal. 2014;12:41 10.1186/s12964-014-0041-2 25027767 PMC4140012

[21] Kraft C, Deplazes A, Sohrmann M, Peter M. Mature ribosomes are selectively degraded upon starvation by an autophagy pathway requiring the Ubp3p/Bre5p ubiquitin protease. Nat Cell Biol. 2008;10(5):602–10. 10.1038/ncb1723 18391941

[22] Durcan TM, Tang MY, Perusse JR, Dashti EA, Aguileta MA, McLelland GL, et al USP8 regulates mitophagy by removing K6-linked ubiquitin conjugates from parkin. EMBO J. 2014;33(21):2473–91. 10.15252/embj.201489729 25216678 PMC4283406

[23] Bingol B, Tea JS, Phu L, Reichelt M, Bakalarski CE, Song Q, et al The mitochondrial deubiquitinase USP30 opposes parkin-mediated mitophagy. Nature. 2014;509(7505):370–5. 10.1038/nature13418 24896179

[24] Cornelissen T, Haddad D, Wauters F, Van Humbeeck C, Mandemakers W, Koentjoro B, et al The deubiquitinase USP15 antagonizes Parkin-mediated mitochondrial ubiquitination and mitophagy. Human molecular genetics. 2014. 10.1093/hmg/ddu244 PMC7108632 24852371

[25] Taillebourg E, Gregoire I, Viargues P, Jacomin A-C, Thevenon D, Faure M, et al The deubiquitinating enzyme USP36 controls selective autophagy activation by ubiquitinated proteins. Autophagy. 2012;8(5):767–79. 10.4161/auto.19381 22622177

[26] Scott RC, Schuldiner O, Neufeld TP. Role and regulation of starvation-induced autophagy in the Drosophila fat body. Dev Cell. 2004;7(2):167–78. 15296714 10.1016/j.devcel.2004.07.009

[27] Neufeld TP. Genetic manipulation and monitoring of autophagy in Drosophila. Methods in enzymology. 2008;451:653–67. 10.1016/S0076-6879(08)03236-9 19185744

[28] Asha H, Nagy I, Kovacs G, Stetson D, Ando I, Dearolf CR. Analysis of Ras-induced overproliferation in Drosophila hemocytes. Genetics. 2003;163(1):203–15. 12586708 10.1093/genetics/163.1.203 PMC1462399

[29] Rusten TE, Lindmo K, Juhász G, Sass M, Seglen PO, Brech A, et al Programmed autophagy in the Drosophila fat body is induced by ecdysone through regulation of the PI3K pathway. Dev Cell. 2004;7(2):179–92. 15296715 10.1016/j.devcel.2004.07.005

[30] Pignoni F, Zipursky SL. Induction of Drosophila eye development by decapentaplegic. Development. 1997;124(2):271–8. 9053304 10.1242/dev.124.2.271

[31] Hennig KM, Colombani J, Neufeld TP. TOR coordinates bulk and targeted endocytosis in the Drosophila melanogaster fat body to regulate cell growth. J Cell Biol. 2006;173(6):963–74. 16785324 10.1083/jcb.200511140 PMC1950482

[32] Mukai A, Yamamoto-Hino M, Awano W, Watanabe W, Komada M, Goto S. Balanced ubiquitylation and deubiquitylation of Frizzled regulate cellular responsiveness to Wg/Wnt. EMBO J. 2010;29(13):2114–25. 10.1038/emboj.2010.100 20495530 PMC2905240

[33] Zhang J, Du J, Lei C, Liu M, Zhu AJ. Ubpy controls the stability of the ESCRT-0 subunit Hrs in development. Development. 2014;141(7):1473–9. 10.1242/dev.099564 24574010 PMC3957371

[34] Berlin I, Schwartz H, Nash PD. Regulation of epidermal growth factor receptor ubiquitination and trafficking by the USP8 STAM complex. J Biol Chem. 2010;285(45):34909–21. 10.1074/jbc.M109.016287 20736164 PMC2966105

[35] Hasdemir B, Murphy JE, Cottrell GS, Bunnett NW. Endosomal deubiquitinating enzymes control ubiquitination and down-regulation of protease-activated receptor 2. J Biol Chem. 2009;284(41):28453–66. 10.1074/jbc.M109.025692 19684015 PMC2788894

[36] Niendorf S, Oksche A, Kisser A, Löhler J, Prinz M, Schorle H, et al Essential role of ubiquitin-specific protease 8 for receptor tyrosine kinase stability and endocytic trafficking in vivo. Mol Cell Biol. 2007;27(13):5029–39. 17452457 10.1128/MCB.01566-06 PMC1951504

[37] Mizuno E, Kobayashi K, Yamamoto A, Kitamura N, Komada M. A deubiquitinating enzyme UBPY regulates the level of protein ubiquitination on endosomes. Traffic. 2006;7(8):1017–31. 16771824 10.1111/j.1600-0854.2006.00452.x

[38] Mizuno E, Iura T, Mukai A, Yoshimori T, Kitamura N, Komada M. Regulation of epidermal growth factor receptor down-regulation by UBPY-mediated deubiquitination at endosomes. Mol Biol Cell. 2005;16(11):5163–74. 16120644 10.1091/mbc.E05-06-0560 PMC1266416

[39] Zhou R, Tomkovicz VR, Butler PL, Ochoa LA, Peterson ZJ, Snyder PM. Ubiquitin-specific Peptidase 8 (USP8) Regulates Endosomal Trafficking of the Epithelial Na+ Channel. J Biol Chem. 2013;288(8):5389–97. 10.1074/jbc.M112.425272 23297398 PMC3581384

[40] Nezis IP, Shravage BV, Sagona AP, Lamark T, Bjørkøy G, Johansen T, et al Autophagic degradation of dBruce controls DNA fragmentation in nurse cells during late Drosophila melanogaster oogenesis. J Cell Biol. 2010;190(4):523–31. 10.1083/jcb.201002035 20713604 PMC2928014

[41] Klionsky DJ, Abdalla FC, Abeliovich H, Abraham RT, Acevedo-Arozena A, Adeli K, et al Guidelines for the use and interpretation of assays for monitoring autophagy. Autophagy. 2012;8(4):445–544. 22966490 10.4161/auto.19496 PMC3404883

[42] Nezis IP, Simonsen A, Sagona AP, Finley K, Gaumer S, Contamine D, et al Ref(2)P, the Drosophila melanogaster homologue of mammalian p62, is required for the formation of protein aggregates in adult brain. J Cell Biol. 2008;180(6):1065–71. 10.1083/jcb.200711108 18347073 PMC2290837

[43] Bartlett BJ, Isakson P, Lewerenz J, Sanchez H, Kotzebue RW, Cumming RC, et al p62, Ref(2)P and ubiquitinated proteins are conserved markers of neuronal aging, aggregate formation and progressive autophagic defects. Autophagy. 2011;7(6):572–83. 21325881 10.4161/auto.7.6.14943 PMC3127048

[44] Carre-Mlouka A, Gaumer S, Gay P, Petitjean AM, Coulondre C, Dru P, et al Control of sigma virus multiplication by the ref(2)P gene of Drosophila melanogaster: an in vivo study of the PB1 domain of Ref(2)P. Genetics. 2007;176(1):409–19. 17409092 10.1534/genetics.106.063826 PMC1893033

[45] Xia R, Jia H, Fan J, Liu Y, Jia J. USP8 promotes smoothened signaling by preventing its ubiquitination and changing its subcellular localization. PLoS Biol. 2012;10(1):e1001238 10.1371/journal.pbio.1001238 22253573 PMC3254663

[46] Lee CY, Baehrecke EH. Steroid regulation of autophagic programmed cell death during development. Development. 2001;128(8):1443–55. 11262243 10.1242/dev.128.8.1443

[47] Gutierrez MG, Munafó DB, Berón W, Colombo MI. Rab7 is required for the normal progression of the autophagic pathway in mammalian cells. J Cell Sci. 2004;117(Pt 13):2687–97. 15138286 10.1242/jcs.01114

[48] Saftig P, Klumperman J. Lysosome biogenesis and lysosomal membrane proteins: trafficking meets function. Nature reviews Molecular cell biology. 2009;10(9):623–35. 10.1038/nrm2745 19672277

[49] Joubert PE, Meiffren G, Gregoire IP, Pontini G, Richetta C, Flacher M, et al Autophagy induction by the pathogen receptor CD46. Cell Host Microbe. 2009;6(4):354–66. 10.1016/j.chom.2009.09.006 19837375

[50] Sarkar S, Korolchuk V, Renna M, Winslow A, Rubinsztein DC. Methodological considerations for assessing autophagy modulators: a study with calcium phosphate precipitates. Autophagy. 2009;5(3):307–13. 19182529 10.4161/auto.5.3.7664

[51] Li S, Chen Y, Shi Q, Yue T, Wang B, Jiang J. Hedgehog-regulated ubiquitination controls smoothened trafficking and cell surface expression in Drosophila. PLoS Biol. 2012;10(1):e1001239 10.1371/journal.pbio.1001239 22253574 PMC3254653

[52] Hans F, Fiesel FC, Strong JC, Jackel S, Rasse TM, Geisler S, et al UBE2E ubiquitin-conjugating enzymes and ubiquitin isopeptidase Y regulate TDP-43 protein ubiquitination. J Biol Chem. 2014;289(27):19164–79. 10.1074/jbc.M114.561704 24825905 PMC4081952

[53] Luo W, Li Y, Tang CH, Abruzzi KC, Rodriguez J, Pescatore S, et al CLOCK deubiquitylation by USP8 inhibits CLK/CYC transcription in Drosophila. Genes Dev. 2012;26(22):2536–49. 10.1101/gad.200584.112 23154984 PMC3505823

[54] Pircs K, Nagy P, Varga A, Venkei Z, Erdi B, Hegedus K, et al Advantages and limitations of different p62-based assays for estimating autophagic activity in Drosophila. PLoS One. 2012;7(8):e44214 10.1371/journal.pone.0044214 22952930 PMC3432079

[55] Scott RC, Juhász G, Neufeld TP. Direct induction of autophagy by Atg1 inhibits cell growth and induces apoptotic cell death. Curr Biol. 2007;17(1):1–11. 17208179 10.1016/j.cub.2006.10.053 PMC1865528

