# Supplementary figures and images for: The Deubiquitinating Enzyme UBPY Is Required for Lysosomal Biogenesis and Productive Autophagy in Drosophila
Source: PLoS One. 2015 Nov 16;10(11):e0143078. doi: 10.1371/journal.pone.0143078 (PMC4646453; doi:10.1371/journal.pone.0143078)

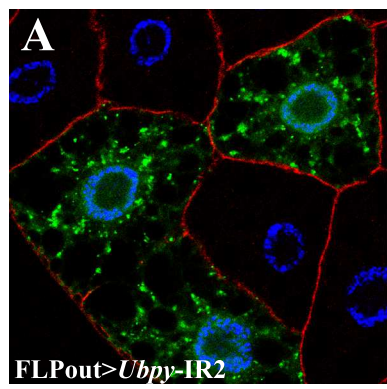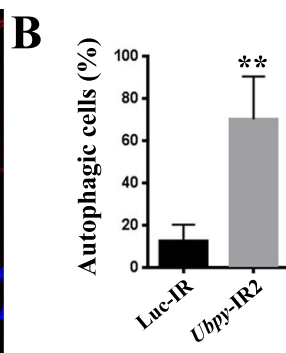

Supplement: S1 Fig — (A) Clonal analysis of a second independant RNAi line targeting Ubpy (Mukai et al 2010) using the FLPout method. Actin is labelled with Phalloidin-Texas Red (red) and nuclei are labelled with Hoechst (blue). Scale bar: 10μm. (B) Quantification of autophagy after silencing by the FLPout method. Bars denote the proportion of autophagic cells from at least 6 animals. Statistical significance was determined using one-way ANOVA: **p<0.005. Genotypes: (A) y w hs-FLP/+; UAS-GFP-Atg8a/+; Ac>CD2>Gal4/ UAS-Ubpy-IR2. (PDF) [file pone.0143078.s001.pdf]

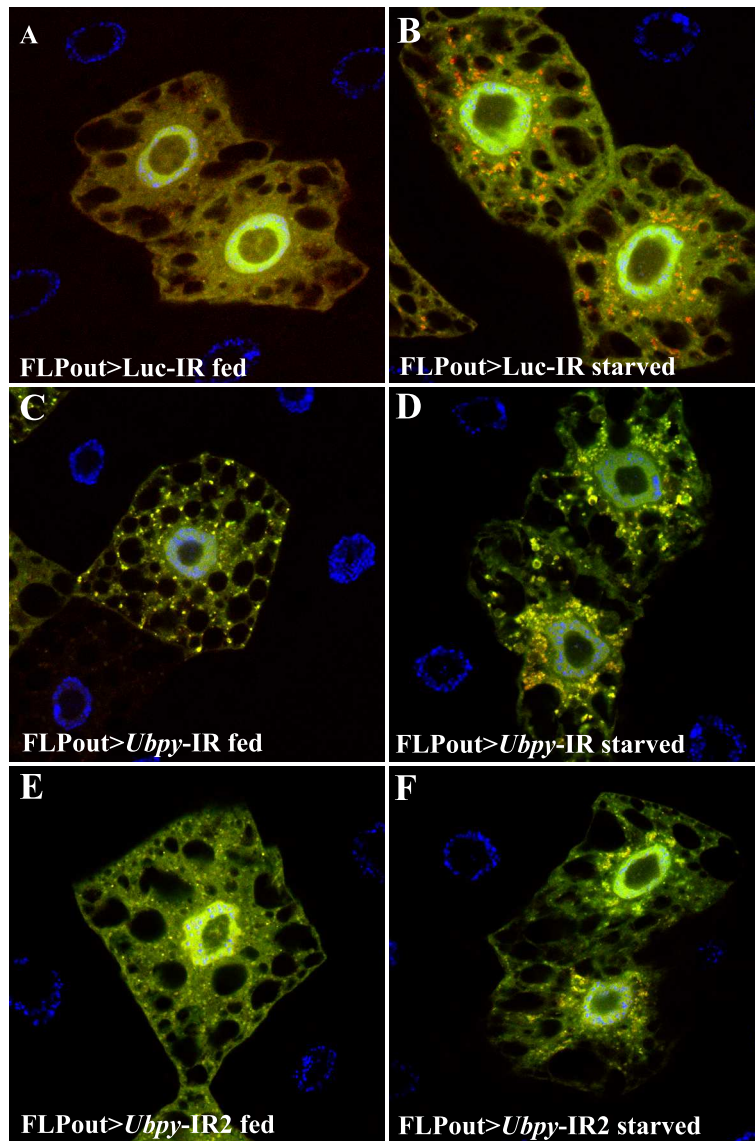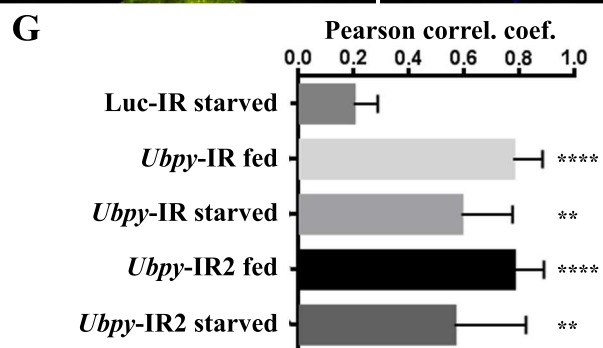

Supplement: S2 Fig — Analysis of the autophagy flux using the tandem-tagged GFP-mCherry-Atg8a reporter in control larvae (A,B) or in Ubpy silenced cells (C-F). Quantification of the colocalization of mCherry and GFP signals using the Pearson’s correlation coefficient is shown in G. N>6 larvae per experimental condition. For all the quantifications, bars denote mean ± s.d. Statistical significance was determined using one-way ANOVA: *p<0.05, **p<0.005, ****p<0.0001. Genotypes: (A, B) y w hs-FLP/+; UAS-GFP-mCherry-Atg8a/UAS-Luc-IR; Ac>CD2>Gal4/+, (C, D) y w hs-FLP/+; UAS-GFP-mCherry-Atg8a/+; Ac>CD2>Gal4/ UAS-Ubpy-IR, (E, F) y w hs-FLP/+; UAS-GFP-mCherry-Atg8a/+; Ac>CD2>Gal4/ UAS-Ubpy-IR2. (PDF) [file pone.0143078.s002.pdf]

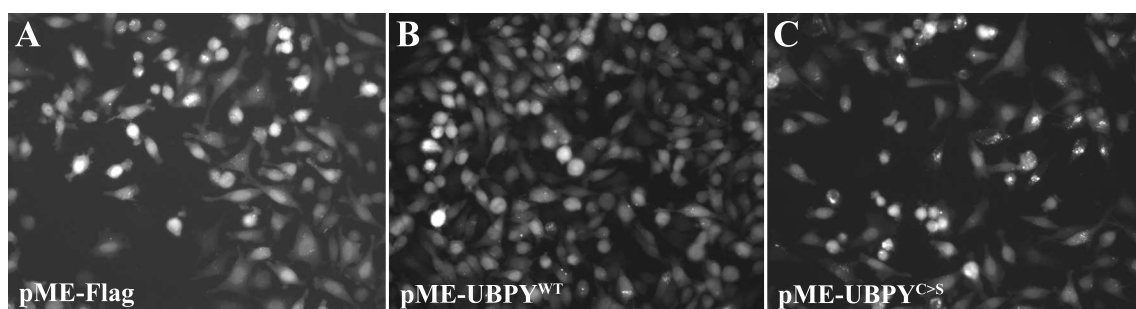

Supplement: S3 Fig — HeLa cells stably expressing the autophagy reporter GFP-LC3 were transfected with a control plasmid (pME-Flag, A) or plasmids expressing either the wild-type human UBPY protein (pME-UBPYWT, B) or its catalytically inactive mutant (pME-UBPYC>S, C). (PDF) [file pone.0143078.s003.pdf]

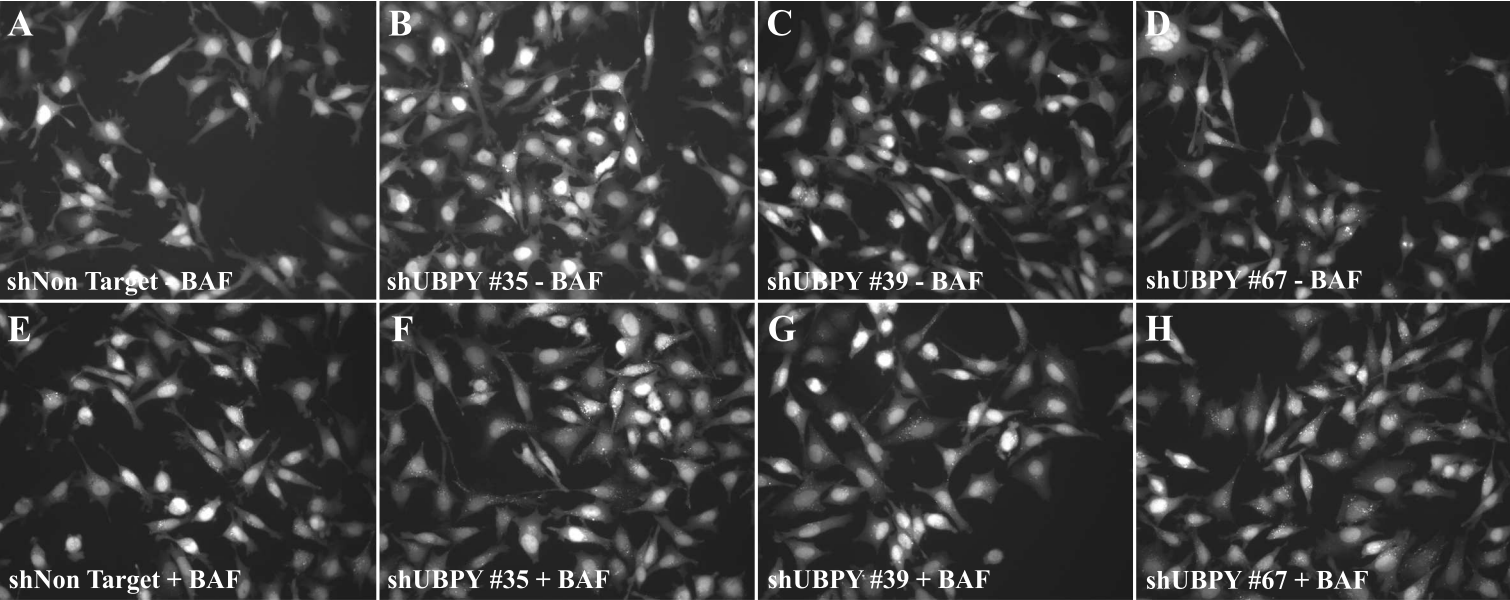

Supplement: S4 Fig — GFP-LC3 HeLa cells were stably transfected with a control shRNA (A, E) or three different shRNAs targeting UBPY (B-D, F-G) in absence (A-D) or in presence of bafilomycin A1 (BAF, E-H). (PDF) [file pone.0143078.s004.pdf]

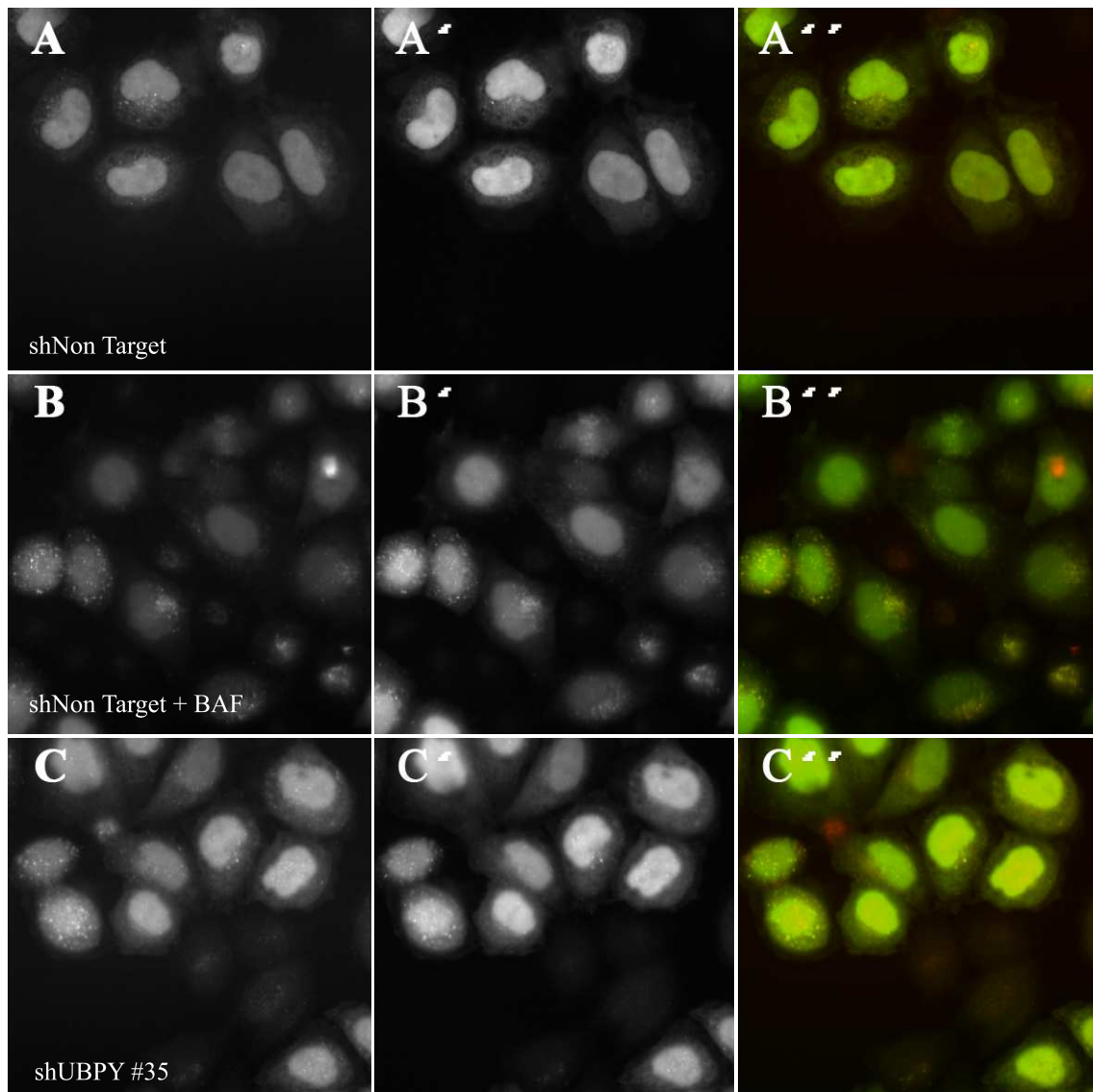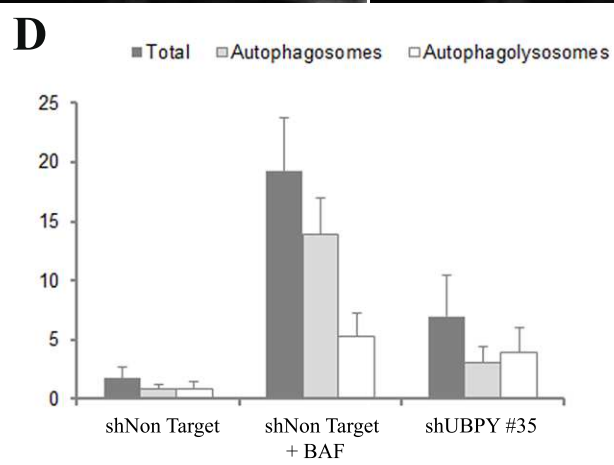

Supplement: S5 Fig — mRFP-GFP-LC3 HeLa cells were stably transfected with either the control shRNA (A, B) or the shUBPY #35 shRNA (C), in comparison with control transfected cells treated with bafilomycin A1 (B). mRFP (A-C), GFP (A’-C’), merge (A”-C”). (D) Quantification of autophagosomes and autolysosomes. (PDF) [file pone.0143078.s005.pdf]
