# Supplementary material for: The Deubiquitinating Enzyme UBPY Is Required for Lysosomal Biogenesis and Productive Autophagy in Drosophila
Source: PLoS One. 2015 Nov 16;10(11):e0143078. doi: 10.1371/journal.pone.0143078 (PMC4646453; doi:10.1371/journal.pone.0143078)
Supplement: S1 Table — List of the phenotypes associated with the fat body specific silencing (using the Cg-GAL4 driver) of the USPs and UCHs tested in this study. Please note that the DUB encoded by the gene CG5505 also known as scrawny or dUsp36 has not been included in this study because its role in autophagy has already been characterized [25]. (PDF) [file pone.0143078.s006.pdf]

1. Phenotypes induced by DUBs silencing in the whole larval fat body.

	Gene Identifier	Symbol	Closest Human DUB	VDRC ID	Strongest phenotype		
					Larval Death	Pupal Death	Autophagic cells
**USPs**	CG15817		USP1	41605			2.85%
	CG14619		USP2				2.22%
	CG12082		USP5	17567		✓	3.085%
	CG1490	USP7	USP7	18281			1.565%
	CG5798	UBPY	USP8	8931		✓	53.705%
	CG1945	faf	USP9X	2955			4.595%
	CG32479		USP10	37858	✓		
	CG7023	USP12	USP12	27799		✓	69.56%
	CG5384		USP14	27405			1.945%
	CG8494		USP20	28910			1%
	CG4166	Not	USP22	45775	✓		
	CG3016		USP30	7090			2.39%
	CG30421		USP31	33726			2.495%
	CG8334		USP32	18981			26.925%
	CG5794		USP34	27517			15.81%
	CG8830		USP38	28960	✓		
	CG7288		USP39	47664			8.145%
	CG4165		USP45	41976			85.585%
	CG5486	Ubp64E	USP47	26027			4.49%
	CG2904	ec	USP54	26027			0.96%
	CG5603	CYLD	CYLD	15340			5.26%
**UCHs**	CG8445	Calypso	BAP1	107757			2.78%
	CG4265	Uch	UCHL3	26468			0%
	CG3431	Uch-L3	UCHL5	34617			65.655%
